# Sensory deficiencies affect resource selection and associational effects at two spatial scales

**DOI:** 10.1002/ece3.4534

**Published:** 2018-10-03

**Authors:** Thomas A. Verschut, Brian D. Inouye, Peter A. Hambäck

**Affiliations:** ^1^ Department of Ecology, Environment and Plant Sciences Stockholm University Stockholm Sweden; ^2^ Department of Biological Science Florida State University Tallahassee Florida

**Keywords:** associational effects, *Drosophila melanogaster*, oviposition, patch selection, resource selection, search behavior

## Abstract

Many insect species have limited sensory abilities and may not be able to perceive the quality of different resource types while approaching patchily distributed resources. These restrictions may lead to differences in selection rates between separate patches and between different resource types within a patch, which may have consequences for associational effects between resources. In this study, we used an oviposition assay containing different frequencies of apple and banana substrates divided over two patches to compare resource selection rates of wild‐type *Drosophila melanogaster* at the between‐ and within‐patch scales. Next, we compared the wild‐type behavior with that of the olfactory‐deficient strain *Orco* ^2^ and the gustatory‐deficient strain *Poxn* ^ΔM22‐B5^ and found comparable responses to patch heterogeneity and similarly strong selection rates for apple at both scales for the wild‐type and olfactory‐deficient flies. Their oviposition behavior translated into associational susceptibility for apple and associational resistance for banana. The gustatory‐deficient flies, on the other hand, no longer had a strong selection rate for apple, strongly differed in between‐ and within‐patch selection rates from the wild‐type flies, and caused no associational effects between the resources. Our study suggests that differences in sensory capabilities can affect resource selection at different search behavior scales in different ways and in turn underlie associational effects between resources at different spatial scales.

## 1 INTRODUCTION

Finding patchily distributed resources can be a challenging task for insects and typically involves decisions at several spatial scales (Cattarino, McAlpine, & Rhodes, 2016; Vinson, 1976; Webster & Cardé, 2017). If an insect is not already within a patch, it first has to locate a suitable patch before selecting a particular resource to consume (Bukovinszky, Potting, Clough, van Lenteren, & Vet, 2005; Stephens & Krebs, 1986; Verschut, Becher, Anderson, & Hambäck, 2016). In the case of polyphagous insects, patch suitability assessment often depends on the quality of multiple resources within the patch rather than the presence of single resource types, which can lead to situations where the use of specific resources depends on the relative frequency of these resources between patches (Hambäck, Inouye, Andersson, & Underwood, 2014; Underwood, Inouye, & Hambäck, 2014). A typical pattern is that the use of less detectable or low‐quality resources is higher when they co‐occur in patches with more detectable or high‐quality resources, which is a phenomenon referred to as associational susceptibility (Letourneau, 1995; Underwood et al., 2014). When the attack rates on the more detectable or high‐quality resources are lower in the same situation, we refer to this situation as associational resistance (Hambäck et al., 2014; Hjältén, Danell, & Lundberg, 1993; Tahvanainen & Root, 1972).

These types of frequency‐dependent resource selection rates have been observed in a range of systems, and it has been suggested that limited sensory capabilities to distinguish between resources types underlies the patterns (Hambäck & Beckerman, 2003; Stephens & Krebs, 1986; Verschut et al., 2016). In insects, it is generally assumed that foraging decisions rely on olfactory or visual cues from longer distances (Burger, Dötterl, & Ayasse, 2010; Murlis, Elkinton, & Cardé, 1992; Saxena, Natesan, & Sane, 2018), and on additional gustatory or mechanosensory cues when selecting between resources that are very close in space (Ozaki et al., 2011; Verschut, Carlsson, Anderson, & Hambäck, 2017). Because different cues may diverge in their information about resource quality (Joseph, Devineni, King, & Heberlein, 2009; Webster & Cardé, 2017), assessments of resource suitability at different scales may not necessarily be correlated. For example, the resolution of sensory information used to locate a resource patch may not be sensitive enough to select between individual resources once an insect has reached the patch (Murlis et al., 1992; Schäpers, Carlsson, Gamberale‐Stille, & Janz, 2015; Webster & Cardé, 2017). As a consequence, the insects may have misinterpreted the actual quality of the immediate resource neighborhood (Hambäck & Beckerman, 2003; Hjältén et al., 1993; Stephens & Krebs, 1986) and make decisions that cause associational effects between different resources.

Considerable neurosensory and molecular research has aimed to functionally characterize specific sensory modalities and the genes regulating sensory neurons (Martín & Alcorta, 2017). This knowledge also holds potential for studies on foraging decisions in heterogeneous environments and can help in exploring the mechanisms underlying frequency‐dependent resource selection rates and associational effects. In two recent papers, we showed that resource finding and oviposition by the fruit fly *Drosophila melanogaster* Meigen (Diptera: Drosophilidae) depend on the frequency of different resources (Verschut, Carlsson et al., 2017; Verschut, Hambäck, & Anderson, 2017). By using mutant *D. melanogaster* strains, deficient in either olfactory or gustatory capabilities, we showed that these sensory deficiencies affect the role of alternative resources during oviposition in different ways. Changes in oviposition behavior were most pronounced for flies lacking the capacity to sense gustatory cues by using gustatory sensilla, which caused females to distribute their eggs independently of resource frequencies within a single patch (Verschut, Carlsson et al., 2017). What we were not able to address in our previous work is how the use of different sensory modalities can underlie associational effects in heterogeneous environments where organisms have to make decisions between and within patches at different spatial scales.

In this paper, we expand our interest from environments with single patches to environments with two patches, allowing us to test how resource selection at two spatial scales depends on sensory modalities. We compare the oviposition rate of wild‐type *D. melanogaster* with that of olfactory and gustatory‐deficient strains in environments where apple and banana oviposition substrates, as two alternative resources, are arranged in separate patches that differ in resource frequencies at the between‐ and within‐patch scales. Furthermore, to compare the strength of resource selection at the two scales, we modified a general resource selection model of Manly (1973) to include hierarchical resource selection. To understand the role of patch complexity, we varied the distribution of apple and banana oviposition substrates to create environments in which the two resources were evenly distributed over two patches, and environments in which one of the two resources was concentrated within a single patch. We hypothesized that wild‐type flies would distinguish between resources at both the between‐ and within‐patch scales. The olfactory‐deficient flies were expected to retain their capacity to select between apple and banana oviposition substrates within patches, but not necessarily have the ability to select between patches. In contrast, we expected gustatory‐deficient flies to lose the capacity to select alternative oviposition substrates within patches, similar as to our previous experiments (Verschut, Carlsson et al., 2017), but they would show some capacity to select between patches through use of longer‐range olfactory cues.

## 2 MATERIAL AND METHODS

### 2.1 Fly rearing and oviposition experiment

We compared the oviposition behavior of wild‐type (*w* ^1118^), olfactory‐deficient (*Orco* ^2^), and gustatory‐deficient (*Poxn* ^ΔM22‐B5^) *Drosophila melanogaster* Meigen (Diptera: Drosophilidae) strains. All flies were reared under controlled conditions (25°C, 50% RH, 12:12 L:D) in 28.5 × 95 mm rearing vials on a standard diet containing corn syrup (115 ml/L), yeast (26 g/L), soy flour (15 g/L), cornmeal (110 g/L), agar (8.5 g/L), and propionic acid (7 ml/L). In the olfactory‐deficient flies, the *Orco* (odorant receptor co‐receptor) mutation effectively silences all odorant receptors by disrupting the heteromeric complex formed by ligand‐binding odorant receptors and their chaperon co‐receptors (Larsson et al., 2004). While the *Orco* mutation severely reduces their sense of smell, the *Orco* ^2^ flies are not fully anosmic as a second family of odorant receptors, called ionotropic receptors, still functions in these flies (Abuin et al., 2011; Benton, Vannice, Gomez‐Diaz, & Vosshall, 2009). The *Poxn* (Pox neuro) mutation leads to a gustatory deficiency by turning all poly‐innervated gustatory bristles on the fly's appendages into mono‐innervated mechanosensory bristles, eliminating all direct contact with the oviposition substrates through the gustatory sensilla (Boll & Noll, 2002).

**Figure 1:**
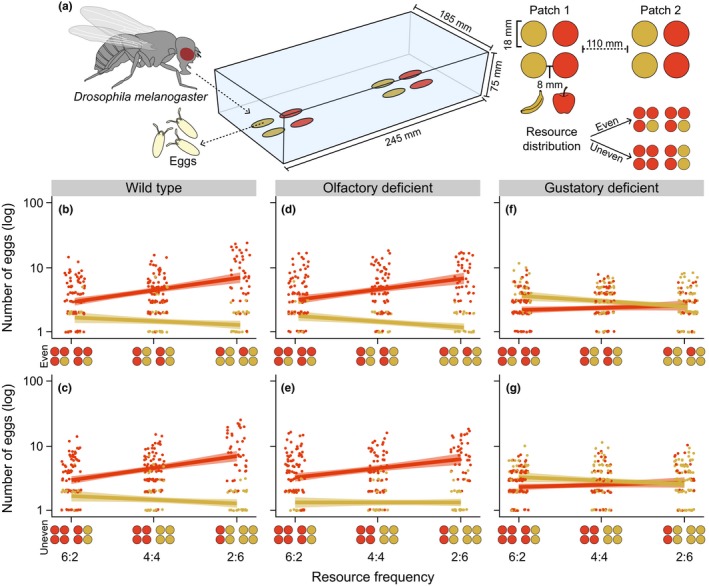
A graphic representation of the oviposition assay and the different resource distribution treatments is given in panel (a), followed by the oviposition behavior of wild‐type *Drosophila melanogaster* (*w* ^1118^ ‐ b,c), olfactory‐deficient flies (*Orco* ^2^ ‐ d,e), and gustatory‐deficient flies (*Poxn* ^ΔM22‐B5^ ‐ f,g) in relation to the increasing frequency of banana oviposition substrates in the oviposition assay. Each point in the graph represents the number of eggs laid by an individual fly on either an apple (red) or banana (yellow) oviposition substrate (log scale) in the oviposition assay with an even resource distribution and an uneven resource distribution. Resource frequency is given as apple:banana on the axis. The predicted linear regression lines for eggs on either resource types are illustrated with their 95% confidence interval, and the points representing the number of eggs laid on a substrate are jittered horizontally for visualization purposes

Three days prior to the experiments, we anesthetized newly eclosed flies with CO_2_ and separated them by sex into 28.5 × 95 mm rearing vials containing the previously described diet, where we let them mature. Approximately an hour before the experiment, we transferred one female and three males into rearing vials to mate. The flies were observed to ensure that mating occurred, and 30 minutes after mating, we transferred the females individually into transparent polypropylene boxes (L × W × H: 245 × 185 × 75 mm) covered with a perforated darkened lid to minimize odor saturation and phototaxis. The boxes contained two patches at the opposite ends separated by a distance of 110 mm (Figure 1a), and each patch consisted of four oviposition substrates that were separated by 8 mm from each other. With this experimental setup, the flies cannot display larger‐scale behavioral processes preceding locating a resource patch; instead, we study how resource heterogeneity affects resource selection between and within patches within a relatively spatially reduced environment. The oviposition substrates consisted of 18‐mm filter paper disks (Grade 1003; Munktell Ahlstrom AB, Sweden) loaded with ripe apple pulp (Discovery, Sweden ‐ 383.80 ± 8.69 mg per disk) or ripe banana pulp (Organic Cavendish ‐ Dole, Dominican Republic ‐ 331.12 ± 7.07 mg per disk) and ensured a standardized and accessible oviposition substrate throughout the experiment. The substrates were placed 15 mm from the edge of the box to ensure that the flies had enough space to walk around the patches.

We created two levels of resource heterogeneity in our oviposition assay. The first level of heterogeneity was represented by the relative frequency of apple and banana in the boxes (hereafter called “resource frequency”), with apple:banana ratios of 6:2, 4:4, and 2:6. The second level of heterogeneity was represented by the distribution of the resources between the two patches within the box (hereafter called “resource distribution”). Resource distribution included even distribution treatments where both patches had an identical number of apple and banana oviposition substrates, and uneven distribution treatments where the resource with the highest frequency was concentrated in one patch and the other patch contained both resource types in the remaining frequencies (see Figure 1a for treatment overview). We ran 20 replicates per patch arrangement and quantified the oviposition behavior by counting the number of eggs laid on each individual oviposition substrate 48 hr after the female was introduced in the oviposition assay, following the protocol of Verschut, Carlsson et al. (2017).

### 2.2 Statistical analysis

We first used generalized linear mixed‐effects models (GLMM) to compare the oviposition patterns of the different strains within the oviposition assay. Afterward, we used a general resource selection model to estimate selection probabilities at the between‐ and within‐patch scales. The distribution of the eggs among the oviposition substrates was analyzed in a GLMM in which each replicate consisted of the numbers of eggs laid by an individual fly on each of the eight oviposition substrates within the oviposition assay. We included resource frequency as a continuous variable accounting for the proportion of banana oviposition substrate in the oviposition assay. We compared the oviposition behavior of the three *D. melanogaster* strains by including strain, resource frequency, resource distribution, resource type, a four‐way interaction, two three‐way interactions based on resource frequency and resource distribution, and all two‐way interactions between the explanatory factors. Because the four‐way interaction was significant but difficult to interpret, we analyzed the oviposition behavior of the individual strains separately in GLMMs including all three‐way and two‐way interactions. All analyses were carried out in R (v. 3.3.2; R Core Team, 2018). The GLMMs were performed using the lme4 package (Bates, Maechler, Bolker, & Walker, 2015), with a negative binomial error distribution, and compared using the car package (Fox & Weisber, 2011) for likelihood ratio tests based on chi‐square values. The ggplot2 package (Wickham, 2016) was used to visualize the oviposition patterns along resource frequency gradients. Model assumptions were checked by estimation of overdispersion and inspections of model residuals.

### 2.3 Estimating between‐ and within‐patch resource selection

To estimate resource selection between and within patches for the *Drosophila* strains with different sensory deficiencies, we expanded the general resource selection model by Manly (1973) to include selection at two scales. We define *N*_*ij*_ as the abundance of resource *i* in patch *j*, where *i* = *A* or *i* = *B* as we only deal with two resources. Resource selection within patches is described by the parameter *s*_*w*_, where *s*_*wa*_ describes the selection of resource *A* and *s*_*wb*_ = (1 − *s*_*wa*_) describes the selection of resource *B*. From this follows that the relative use of resource *A* within a single patch is

(1) $$W_{a}=\frac{s_{\mathrm{wa}}\;\times \;N_{a}}{s_{\mathrm{wa}}\;\times \;N_{a}+\left(1-s_{\mathrm{wa}}\right)\;\times \;N_{b}}$$

where *W*_*b*_ = 1 − *W*_*a*_ is the relative use of resource *B* within a patch. At the between‐patch scale, the influence of resource *i* on patch selection is described by the parameter *s*_*pji*_, where *s*_*pja*_ determines the relative importance of resource *A* while *s*_*pjb*_ = (1 − *s*_*pja*_) determines the relative importance of resource *B* for selecting patch *j*. When we assume *n* patches, which may contain one or both resource types *A* and *B*, the relative use of patch *j* is

(2) $$P_{j}=\frac{s_{\mathrm{pja}}\;\times \;N_{\mathrm{aj}}+\left(1-s_{\mathrm{pja}}\right)\;\times \;N_{\mathrm{bj}}}{s_{\mathrm{pja}}\;\times \;\sum_{j=1}^{n}N_{\mathrm{aj}}+\left(1-s_{\mathrm{pja}}\right)\;\times \;\sum_{j=1}^{n}N_{\mathrm{bj}}}$$

When we combine these equations, assuming that the selection coefficients between (*s*_*p*_) and within (*s*_*w*_) patches are independent, the relative use of resource *A* in patch *j* (with a corresponding function for resource *B*) will be:

(3) $$T_{\mathrm{ai}}=W_{a}\times P_{j}$$

We used the number of eggs laid on each oviposition substrate to calculated maximum likelihood estimates of the model selection coefficients, *s*_*wa*_ and *s*_*pja*_, assuming a binomial likelihood function. In addition to the six treatments shown in Figure 1a, we included data from one additional treatment to improve the estimation of the selection coefficients (see Supporting information Figure S1). While this additional treatment also had an overall 4:4 ratio of the two resource types, the oviposition substrates were arranged such that there were 3:1 and 1:3 ratios of each resource type within each patch. The maximum likelihood estimates and profile confidence intervals were calculated using a range of starting values for the parameter search algorithm in the bbmle package (Bolker, 2017), and the estimates were robust to different starting values for the search algorithm parameters.

## 3 RESULTS

### 3.1 Oviposition experiment

A significant four‐way interaction (χ^2^ = 14.05, *p *=* *0.015; Supporting information Table S1) indicated that interactions between the explanatory factors differed between the strains. To simplify the interpretation of this result, we analyzed the oviposition patterns for the three strains separately (Figure 1; Supporting information Table S2). These analyses indicated a three‐way interaction between resource frequency, resource distribution, and resource type for the olfactory‐deficient flies, but not for the wild‐type or gustatory‐deficient flies (Supporting information Table S2). The interpretation of these differences was apparent when comparing the results for the individual strains. First, wild‐type flies had a strong preference to lay eggs on apple (GLM: χ^2^ = 650.71; *p *<* *0.001; Figure 1b,c), and this preference strongly increased in the treatments where apple was an uncommon resource (i.e., with a high frequency of banana, GLM: χ^2^ = 64.12; *p *<* *0.001; Supporting information Table S2). Second, the olfactory‐deficient flies similarly had a strong preference for apple (GLM: χ^2^ = 621.09, *p *<* *0.001), and this preference was again stronger when apple was less common (GLM: χ^2^ = 44.17, *p *<* *0.001; Figure 1; Supporting information Table S2). The oviposition behavior of both the wild‐type and olfactory‐deficient flies translated into associational susceptibility for apple, as the oviposition rate on apple increased with the increasing frequency of banana in the patch, and into associational resistance for banana, as the oviposition rate on banana decreased with increasing frequency of apple. However, the analysis of the olfactory‐deficient flies also indicated a three‐way interaction between resource frequency, resource distribution, and resource type (GLM: χ^2^ = 7.64, *p *=* *0.005; Supporting information Table S2), which occurred because the oviposition rate on banana decreased more with an increasing frequency of banana in the even resource distribution treatments than in the uneven resource distribution treatments (Figure 1d,e). Finally, for the gustatory‐deficient flies we found a preference for banana (GLM: χ^2^ = 8.56, *p *=* *0.003) that was affected by an interaction between resource type and resource frequency (GLM: χ^2^=20.71, *p *<* *0.001; Supporting information Table S2). The interaction occurred because the preference for banana decreased with the overall banana frequency (Figure 1f,g), resulting in the occurrence of associational susceptibility for banana and no associational effect for apple.

### 3.2 Resource selection between and within patches

**Figure 2:**
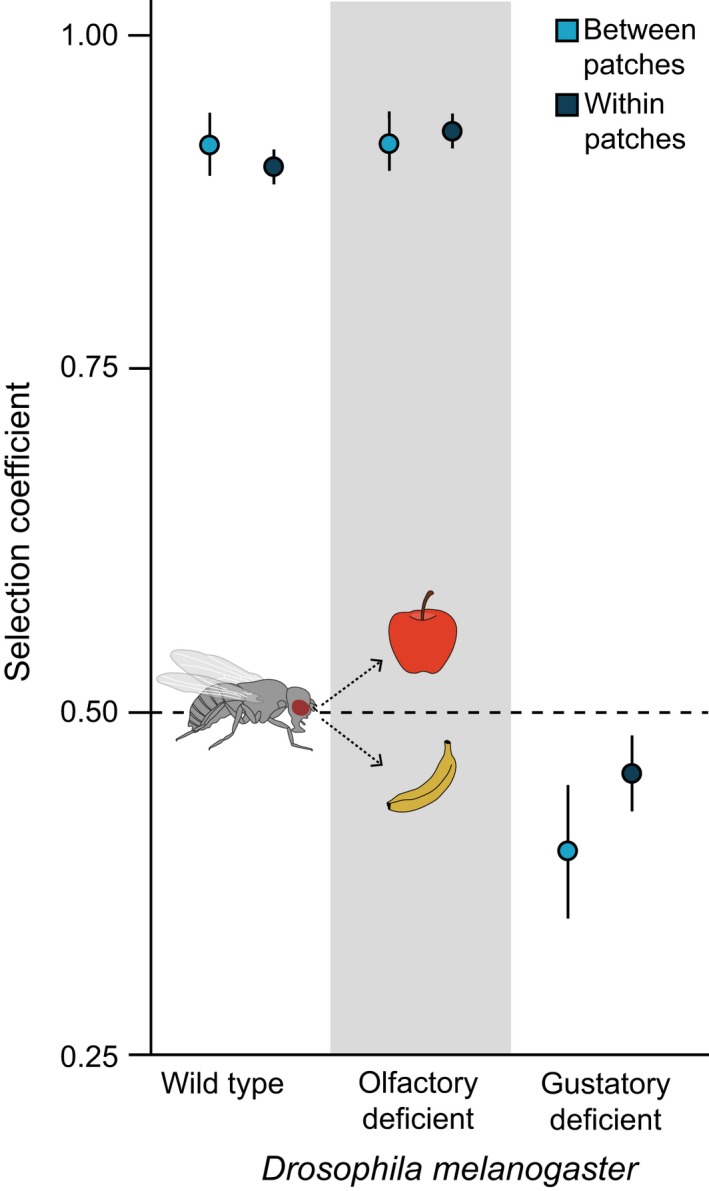
Maximum likelihood estimates for the selection coefficients between patches (light blue) and within patches (dark blue) for wild‐type *Drosophila melanogaster* (*w* ^1118^), olfactory‐deficient flies (*Orco* ^2^), and gustatory‐deficient flies (*Poxn* ^ΔM22‐B5^). The dashed line at 0.50 represents random selection among the apple or banana oviposition substrates. Selection coefficients above the dashed line represent a preference for apple, and selection coefficients below the dashed line represent a preference for banana

The maximum likelihood estimates for the selection coefficients of the wild‐type flies and olfactory‐deficient flies were close to 1.00 and signified a strong preference of these flies to select apple at the between‐patch and within‐patch scales (Supporting information Figure S1). Interestingly, the between‐patch selection coefficients are nearly identical for the wild‐type flies and olfactory‐deficient flies, but the within‐patch selection coefficients have non‐overlapping 95% confidence intervals (Figure 2). The selection coefficients of the gustatory‐deficient flies were below 0.50, indicating a preference for banana. However, the selection coefficient for the within‐patch selection was closer to 0.50 than the between‐patch selection coefficient (Figure 2), indicating a weaker preference at the within‐patch scale.

## 4 DISCUSSION

Our results showed that olfactory and gustatory sensory deficiencies have different effects on the oviposition behavior of *Drosophila melanogaster* in heterogeneous environments. Both wild‐type flies and olfactory‐deficient flies showed strong selection for apple over banana substrates, whereas gustatory‐deficient flies selected banana over apple substrates. Moreover, for wild‐type flies and olfactory‐deficient flies, selection of the preferred apple substrates strongly increased when the frequency of apple decreased (Figure 1), corresponding to associational susceptibility for apple and associational resistance for banana. In contrast, no associational effects were observed for the gustatory‐deficient flies. When comparing selection between and within patches, we found nearly identical resource selection coefficients at the two scales for wild‐type flies (Figure 2). Olfactory‐deficient flies, on the other hand, showed slightly stronger selection within patches compared to between patches, while gustatory‐deficient flies showed stronger selection between patches. These results suggest that the two sensory modalities affected resource selection at the two scales in different ways, and that gustatory cues are more important in causing associational effects at the spatial scale used in the present study.

More specifically, the higher selection of apple by the wild‐type and olfactory‐deficient flies, compared to higher selection of banana by the gustatory‐deficient flies, indicates that the two sensory modalities detect the resources differently. The reason why the gustatory‐deficient flies were more likely to select banana could be their tendency to spend more time walking on the banana substrates than the two other strains during the experiment (T. A. Verschut, personal observation). Therefore, it is possible that the banana substrates contain gustatory cues that lower the probability of the wild‐type and olfactory‐deficient flies from accessing it or laying eggs on it (Schwartz, Zhong, Bellemer, & Tracey, 2012; Yang, Belawat, Hafen, Jan, & Jan, 2008). Under natural conditions, *Drosophila* females usually oviposit on fruits that gradually become richer in nutritional content due to the presence of yeasts (Becher et al., 2012; Lihoreau, Poissonnier, Isabel, & Dussutour, 2016). As the gustatory sensilla are usually needed to fully assess the nutritional content of a resource (Montell, 2009; Shim et al., 2015), it is likely that the gustatory‐deficient flies were impaired in doing so (Depetris‐Chauvin, Galagovsky, & Grosjean, 2015; Joseph et al., 2009).

Previous studies have suggested that both gustatory and mechanosensory cues are important while selecting resource at small spatial scales (Ozaki et al., 2011; Verschut, Carlsson et al., 2017; Zhang, Aikin, Li, & Montell, 2016). In our experiments, the gustatory‐deficient flies had lost their ability to assess the differences between the two substrates at small scales through direct contact with the oviposition substrates (Joseph et al., 2009; Verschut, Carlsson et al., 2017), but likely retained their ability to selected resources at larger scales through the use of olfaction (Hussain et al., 2016; Lin, Prokop‐Prigge, Preti, & Potter, 2015). As we found that these flies showed stronger selection between patches, our results comply with the expectation that gustatory deficiencies would mainly affect resource selection at the within‐patch scale. The stronger selection for banana at the between‐patch scale could be a result of the olfactory attraction of adult flies to banana odors as a food source (Lachaise & Silvain, 2004; Schubert, Hansson, & Sachse, 2014), making it is possible that the oviposition choice of the gustatory‐deficient flies was influenced by the selection of adult food resources at the between‐patch scale (Jaenike, 1982; Scheirs & De Bruyn, 2002; Wiklund, 1975). Similar to what we found in a previous study (Verschut, Carlsson et al., 2017), the changes in resource preference by the gustatory‐deficient flies caused an absence of the associational effects (Figure 1). In Verschut, Carlsson et al. (2017), we found that the loss of gustatory sensilla not only resulted in random selection between resources, but also eliminated associational susceptibility for apple and associational resistance for banana within a single patch. As our current results show that gustatory deficiency also has strong effects on resource selection at the two spatial scales of our experiment, we can expect that gustatory deficiencies will eliminate associational effects at different spatial scales in natural environments.

We also expected that the olfactory‐deficient flies would retain their ability to select between the resources within the patches but have lower abilities to select between patches, and indeed, there was a slightly weaker selection between patches by these flies. However, the slight difference in selection rates suggests that the olfactory deficiency had no strong effect on the ability to assess the differences between the two resources at the two scales of our oviposition assay. As we only found slight differences in the between‐ and within‐patch selection rates for both the wild‐type and olfactory‐deficient flies, we assume that the overall spatial scale of our experiment may have been too small for olfactory cues to be of importance. Several larger‐scale studies have shown that resource selection through olfaction may be a process that already occurs, while *D. melanogaster* is flying toward odor source (Becher, Bengtsson, Hansson, & Witzgall, 2010; Lebreton, Becher, Hansson, & Witzgall, 2012; Verschut, Hambäck et al., 2017). Moreover, the lack of differences between selection rates may also be a result of short‐range olfactory preference for fruit substrates being mediated through a single olfactory receptor channel in which apple and banana odors induce very similar physiological responses (Dweck et al., 2013). Consequently, a fully functioning repertoire of olfactory receptors may not have provided the wild‐type flies with additional short‐range information, relevant for their oviposition choice, compared to what the olfactory‐deficient flies perceived.

While the restricted size of our oviposition assay minimized the ability of the flies to select resources through olfaction, we did find a stronger decrease in the oviposition rate on banana with an increasing frequency of banana in the even resource distribution treatments than in the uneven resource distribution treatments (Figure 1d,e; Supporting information Table S2). This might have been a result of the *Orco* ^2^ olfactory‐deficient flies not being fully anosmic (Larsson et al., 2004), as a second family of odorant receptors, called ionotropic receptors, still functions in these flies (Abuin et al., 2011; Benton et al., 2009). In fact, Silbering et al. (2011) showed that, in comparison with wild‐type flies, *Orco* mutant flies have a stronger aversion to odors associated with carboxylic acids and an increased attraction to amines. The small changes in the evaluation of these odors may have caused the olfactory‐deficient flies to show slight differences in their oviposition behavior in the even and uneven resource distribution treatments. While we do not have data on the odors present in our substrates, these types of odorants are commonly found at oviposition sites of *D. melanogaster* (Mansourian & Stensmyr, 2015; Silbering et al., 2011), and we expect that the use of the ionotropic receptors has allowed the olfactory‐deficient flies to remain their capabilities to strongly select for apple at the between‐ and within‐patch scale. In a previous study, we actually found that mutations in the ionotropic co‐receptors responsible for sensing carboxylic acids and amines do not change *D. melanogaster's* preference to select apple over banana in single patch oviposition assays (Verschut, Carlsson et al. 2017), suggesting that deficiencies in the olfactory system play a minimal role in selection resources at the scales tested in our oviposition assays. Consequently, our results suggest that the use of gustatory cues is sufficient to select the appropriate oviposition resources at the two scales used in our oviposition assay.

The prevailing hypothesis about associational effects suggests that misinterpretation of resource quality while selecting resources at different scales is the main reason for its occurrence (Hambäck et al., 2014; Hjältén et al., 1993). Consequently, the scale at which factors like plant apparency (Castagneyrol, Giffard, Péré, & Jactel, 2013; Verschut et al., 2016), defense traits (Sato, Ito, & Kudoh, 2017; Sato & Kudoh, 2015), or palatability (Hahn & Orrock, 2016; Kim, Underwood, & Inouye, 2013) are distinguished may directly affect resource selection at the between‐ and within‐patch scales and have direct consequences for associational effects. While we previously found that the relative frequency of different resources at the within‐patch scale is sufficient to cause associational effects when insects strongly select between two resources within a patch (Verschut, Carlsson et al., 2017; Verschut, Hambäck, & Anderson, 2017), our current study suggests that nearly identical selection behavior at the between‐ and within‐patch scales can underlie the occurrence of associational effects in environments with multiple patches. However, other studies have suggested that oviposition choices under natural conditions may occur at even larger scales (Janz, Bergström, & Johansson, 2005; Wise, Yi, & Abrahamson, 2009), potentially affecting both associational effects and resource coexistence at larger scales (Barbosa et al., 2009; Underwood et al., 2014). While it is of interest to test our hypothesis at larger spatial scales, our current results suggest that the degree to which an insect is able to distinguish between resources through different sensory modalities will affect the pattern of associational effects at different spatial scales.

## CONFLICT OF INTEREST

None declared.

## AUTHOR CONTRIBUTION

TAV and PAH conceived the study. TAV performed the oviposition experiments. TAV, BDI, and PAH performed the statistical modeling analysis. TAV, BDI, and PAH wrote the manuscript.

## DATA ACCESSIBILITY

Data files supporting this study are available in the Dryad Digital Repository:https://doi.org/10.5061/dryad.tk033rk.

## COMPETING FINANCIAL INTERESTS

The authors declare no competing financial interests.

## References

[ece34534-bib-0001] Abuin, L. , Bargeton, B. , Ulbrich, M. H. , Isacoff, E. Y. , Kellenberger, S. , & Benton, R. (2011). Functional architecture of olfactory ionotropic glutamate receptors. Neuron, 69, 44–60. 10.1016/j.neuron.2010.11.042 21220098 PMC3050028

[ece34534-bib-0002] Barbosa, P. , Hines, J. , Kaplan, I. , Martinson, H. , Szczepaniec, A. , & Szendrei, Z. (2009). Associational resistance and associational susceptibility: Having right or wrong neighbors. Annual Review of Ecology, Evolution, and Systematics, 40, 1–20. 10.2307/20744029

[ece34534-bib-0003] Bates, D. , Maechler, M. , Bolker, B. , & Walker, S. (2015). Fitting linear mixed‐effects models using lme4. Journal of Statistical Software, 67, 1–48. 10.18637/jss.v067.i01

[ece34534-bib-0004] Becher, P. G. , Bengtsson, M. , Hansson, B. S. , & Witzgall, P. (2010). Flying the fly: Long‐range flight behavior of *Drosophila melanogaster* to attractive odors. Journal of Chemical Ecology, 36, 599–607. 10.1007/s10886-010-9794-2 20437263

[ece34534-bib-0005] Becher, P. G. , Flick, G. , Rozpedowska, E. , Schmidt, A. , Hagman, A. , Lebreton, S. , … Bengtsson, M. (2012). Yeast, not fruit volatiles mediate *Drosophila melanogaster* attraction, oviposition and development. Functional Ecology, 26, 822–828. 10.1111/j.1365-2435.2012.02006.x

[ece34534-bib-0006] Benton, R. , Vannice, K. S. , Gomez‐Diaz, C. , & Vosshall, L. B. (2009). Variant ionotropic glutamate receptors as chemosensory receptors in *Drosophila* . Cell, 136, 149–162. 10.1016/j.cell.2008.12.001 19135896 PMC2709536

[ece34534-bib-0007] Bolker, B. (2017) bbmle: tools for general maximum likelihood estimation ‐ R package version 1.0.20. https://CRAN.R-project.org/package=bbmle.

[ece34534-bib-0008] Boll, W. , & Noll, M. (2002). The *Drosophila Pox neuro* gene: Control of male courtship behavior and fertility as revealed by a complete dissection of all enhancers. Development, 129, 5667–5681. 10.1242/dev.00157 12421707

[ece34534-bib-0009] Bukovinszky, T. , Potting, R. P. J. , Clough, Y. , van Lenteren, J. C. , & Vet, L. E. M. (2005). The role of pre‐ and post‐ alighting detection mechanisms in the responses to patch size by specialist herbivores. Oikos, 109, 435–446. 10.1111/j.0030-1299.2005.13707.x

[ece34534-bib-0010] Burger, H. , Dötterl, S. , & Ayasse, M. (2010). Host‐plant finding and recognition by visual and olfactory floral cues in an oligolectic bee. Functional Ecology, 24, 1234–1240. 10.1111/j.1365-2435.2010.01744.x

[ece34534-bib-0011] Castagneyrol, B. , Giffard, B. , Péré, C. , & Jactel, H. (2013). Plant apparency, an overlooked driver of associational resistance to insect herbivory. Journal of Ecology, 101, 418–429. 10.1111/1365-2745.12055

[ece34534-bib-0012] Cattarino, L. , McAlpine, C. A. , & Rhodes, J. R. (2016). Spatial scale and movement behaviour traits control the impacts of habitat fragmentation on individual fitness. Journal of Animal Ecology, 85, 168–177. 10.1111/1365-2656.12427 26250334

[ece34534-bib-0013] Depetris‐Chauvin, A. , Galagovsky, D. , & Grosjean, Y. (2015). Chemicals and chemoreceptors: Ecologically relevant signals driving behavior in *Drosophila* . Frontiers in Ecology and Evolution, 3, 41 10.3389/fevo.2015.00041

[ece34534-bib-0014] Dweck, H. K. M. , Ebrahim, S. A. M. , Kromann, S. H. , Bown, D. , Hillbur, Y. , Sachse, S. , … Stensmyr, M. C. (2013). Olfactory preference for egg laying on citrus substrates in *Drosophila* . Current Biology, 23, 1–9. 10.1016/j.cub.2013.10.047 24316206

[ece34534-bib-0015] Fox, J. , & Weisber, S. (2011). An R companion to applied regression. Thousand Oaks, California: Sage Publications.

[ece34534-bib-0016] Hahn, P. G. , & Orrock, J. L. (2016). Neighbor palatability generates associational effects by altering herbivore foraging behavior. Ecology, 97, 2103–2111. 10.1002/ecy.1430 27859184

[ece34534-bib-0017] Hambäck, P. A. , & Beckerman, A. P. (2003). Herbivory and plant resource competition: A review of two interacting interactions. Oikos, 101, 26–37. 10.1034/j.1600-0706.2003.12568.x

[ece34534-bib-0018] Hambäck, P. A. , Inouye, B. D. , Andersson, P. , & Underwood, N. (2014). Effects of plant neighborhoods on plant‐herbivore interactions: Resource dilution and associational effects. Ecology, 95, 1370–1383. 10.1890/13-0793.1 25000768

[ece34534-bib-0019] Hjältén, J. , Danell, K. , & Lundberg, P. (1993). Herbivore avoidance by association: Vole and hare utilization of woody plants. Oikos, 68, 125–131. 10.2307/3545317

[ece34534-bib-0020] Hussain, A. , Zhang, M. , Ápunar, H. K. , Svensson, T. , Quillery, E. , Gompel, N. , … Grunwald Kadow, I. C. (2016). Ionotropic chemosensory receptors mediate the taste and smell of polyamines. PLoS Biology, 14, e1002454 10.1371/journal.pbio.1002454 27145030 PMC4856413

[ece34534-bib-0021] Jaenike, J. (1982). Environmental modification of oviposition behavior in *Drosophila* . The American Naturalist, 119, 784–802. 10.1086/283955

[ece34534-bib-0022] Janz, N. , Bergström, A. , & Johansson, J. (2005). Frequency dependence of host plant choice within and between patches: A large cage experiment. Evolutionary Ecology, 19, 289–302. 10.1007/s10682-005-6078-3

[ece34534-bib-0023] Joseph, R. M. , Devineni, A. V. , King, I. F. G. , & Heberlein, U. (2009). Oviposition preference for and positional avoidance of acetic acid provide a model for competing behavioral drives in *Drosophila* . Proceedings of the National Academy of Sciences of the United States of America, 106, 11352–11357. 10.1073/pnas.0901419106 19541615 PMC2698888

[ece34534-bib-0024] Kim, T. N. , Underwood, N. , & Inouye, B. D. (2013). Insect herbivores change the outcome of plant competition through both inter‐ and intraspecific processes. Ecology, 94, 1753–1763. 10.1890/12-1261.1 24015519

[ece34534-bib-0025] Lachaise, D. , & Silvain, J. F. (2004). How two afrotropical endemics made two cosmopolitan human commensals: The *Drosophila Melanogaster – D. Simulans* palaeogeographic riddle. Genetica, 120, 17–39. 10.1023/B:GENE.0000017627.27537.ef 15088644

[ece34534-bib-0026] Larsson, M. C. , Domingos, A. I. , Jones, W. D. , Chiappe, M. E. , Amrein, H. , & Vosshall, L. B. (2004). *Or83b* encodes a broadly expressed odorant receptor essential for *Drosophila* olfaction. Neuron, 43, 703–714. 10.1016/j.neuron.2004.08.019 15339651

[ece34534-bib-0027] Lebreton, S. , Becher, P. G. , Hansson, B. S. , & Witzgall, P. (2012). Attraction of *Drosophila melanogaster* males to food‐related and fly odours. Journal of Insect Physiology, 58, 125–129. 10.1016/j.jinsphys.2011.10.009 22067291

[ece34534-bib-0028] Letourneau, D. K. (1995). Associational susceptibility: Effects of cropping pattern and fertilizer on Malawian bean fly levels. Ecological Applications, 5, 823–829. 10.2307/1941990

[ece34534-bib-0029] Lihoreau, M. , Poissonnier, L. A. , Isabel, G. , & Dussutour, A. (2016). *Drosophila* females trade off good nutrition with high‐quality oviposition sites when choosing foods. The Journal of Experimental Biology, 219, 2514–2524. 10.1242/jeb.142257 27284071

[ece34534-bib-0030] Lin, C. C. , Prokop‐Prigge, K. A. , Preti, G. , & Potter, C. J. (2015). Food odors trigger *Drosophila* males to deposit a pheromone that guides aggregation and female oviposition decisions. eLife, 4, e08688 https://doi.org/10.7554/eLife. 08688 26422512 10.7554/eLife.08688 PMC4621432

[ece34534-bib-0031] Manly, B. F. J. (1973). A linear model for frequency‐dependent selection by predators. Researches on Population Ecology, 14, 137–150. 10.1007/bf02518839

[ece34534-bib-0032] Mansourian, S. , & Stensmyr, M. C. (2015). The chemical ecology of the fly. Current Opinion in Neurobiology, 34, 95–102. 10.1016/j.conb.2015.02.006 25747730

[ece34534-bib-0033] Martín, F. , & Alcorta, E. (2017). Novel genetic approaches to behavior in *Drosophila* . Journal of Neurogenetics, 31, 288–299. 10.1080/01677063.2017.1395875 29119859

[ece34534-bib-0034] Montell, C. (2009). A taste of the *Drosophila* gustatory receptors. Current Opinion in Neurobiology, 19, 345–353. 10.1016/j.conb.2009.07.001 19660932 PMC2747619

[ece34534-bib-0035] Murlis, J. , Elkinton, J. S. , & Cardé, R. T. (1992). Odor plumes and how insects use them. Annual Review of Entomology, 37, 505–532. 10.1146/annurev.en.37.010192.002445

[ece34534-bib-0036] Ozaki, K. , Ryuda, M. , Yamada, A. , Utoguchi, A. , Ishimoto, H. , Calas, D. , … Yoshikawa, H. (2011). A gustatory receptor involved in host plant recognition for oviposition of a swallowtail butterfly. Nature Communications, 2, 542 10.1038/ncomms1548 22086342

[ece34534-bib-0037] R Core Team (2018). R: A language and environment for statistical computing. Vienna, Austria: R Foundation for Statistical Computing https://www.R-project.org/.

[ece34534-bib-0038] Sato, Y. , Ito, K. , & Kudoh, H. (2017). Optimal foraging by herbivores maintains polymorphism in defence in a natural plant population. Functional Ecology, 31, 2233–2243. https://doi.org/doi:10.1111/1365-2435.12937

[ece34534-bib-0039] Sato, Y. , & Kudoh, H. (2015). Tests of associational defence provided by hairy plants for glabrous plants of *Arabidopsis halleri* subsp. gemmifera against insect herbivores. Ecological Entomology, 40, 269–279. 10.1111/een.12179

[ece34534-bib-0040] Saxena, N. , Natesan, D. , & Sane, S. P. (2018). Odor source localization in complex visual environments by fruit flies. The Journal of Experimental Biology, 221, 10.1242/jeb.172023 29146771

[ece34534-bib-0041] Schäpers, A. , Carlsson, M. A. , Gamberale‐Stille, G. , & Janz, N. (2015). The role of olfactory cues for the search behavior of a specialist and generalist butterfly. Journal of Insect Behavior, 28, 77–87. 10.1007/s10905-014-9482-0

[ece34534-bib-0042] Scheirs, J. , & De Bruyn, L. (2002). Integrating optimal foraging and optimal oviposition theory in plant‐insect research. Oikos, 96, 187–191. 10.1034/j.1600-0706.2002.960121.x

[ece34534-bib-0043] Schubert, M. , Hansson, B. S. , & Sachse, S. (2014). The banana code ‐ natural blend processing in the olfactory circuitry of *Drosophila melanogaster* . Frontiers in Physiology, 5, 59 10.3389/fphys.2014.00059 24600405 PMC3929855

[ece34534-bib-0044] Schwartz, N. U. , Zhong, L. , Bellemer, A. , & Tracey, W. D. (2012). Egg laying decisions in *Drosophila* are consistent with foraging costs of larval progeny. PLoS ONE, 7, e37910 10.1371/journal.pone.0037910 22693584 PMC3365076

[ece34534-bib-0045] Shim, J. , Lee, Y. , Jeong, Y. T. , Kim, Y. , Lee, M. G. , Montell, C. , & Moon, S. J. (2015). The full repertoire of *Drosophila* gustatory receptors for detecting an aversive compound. Nature Communications, 6, 8867 10.1038/ncomms9867 PMC4660205 26568264

[ece34534-bib-0046] Silbering, A. F. , Rytz, R. , Grosjean, Y. , Abuin, L. , Ramdya, P. , Jefferis, G. S. X. E. , & Benton, R. (2011). Complementary function and integrated wiring of the evolutionarily distinct *Drosophila* olfactory subsystems. Journal of Neuroscience, 31, 13357–13375. 10.1523/jneurosci.2360-11.2011 21940430 PMC6623294

[ece34534-bib-0047] Stephens, D. W. , & Krebs, J. R. (1986). Foraging theory. Princeton, NJ: Princeton University Press.

[ece34534-bib-0048] Tahvanainen, J. O. , & Root, R. B. (1972). The influence of vegetational diversity on the population ecology of a specialized herbivore, *Phyllotreta cruciferae* (Coleoptera: Chrysomelidae). Oecologia, 10, 321–346. 10.1007/BF00345736 28307065

[ece34534-bib-0049] Underwood, N. , Inouye, B. D. , & Hambäck, P. A. (2014). A conceptual framework for associational effects: When do neighbors matter and how would we know? Quarterly Review of Biology, 89, 1–19. 10.1086/674991 24672901

[ece34534-bib-0050] Verschut, T. A. , Becher, P. G. , Anderson, P. , & Hambäck, P. A. (2016). Disentangling associational effects: Both resource density and resource frequency affect search behaviour in complex environments. Functional Ecology, 30, 1826–1833. 10.1111/1365-2435.12670

[ece34534-bib-0051] Verschut, T. A. , Carlsson, M. A. , Anderson, P. , & Hambäck, P. A. (2017). Sensory mutations in *Drosophila melanogaster* influence associational effects between resources during oviposition. Scientific Reports, 7, 9352 10.1038/s41598-017-09728-7 28839208 PMC5570953

[ece34534-bib-0052] Verschut, T. A. , Hambäck, P. A. , & Anderson, P. (2017). Mating affects resource selection through olfactory‐guided behaviour and modulates associational effects between neighbouring resources. Oikos, 126, 1708–1716. 10.1111/oik.04315

[ece34534-bib-0053] Vinson, S. B. (1976). Host selection by insect parasitoids. Annual Review of Entomology, 21, 109–133. 10.1146/annurev.en.21.010176.000545

[ece34534-bib-0054] Webster, B. , & Cardé, R. T. (2017). Use of habitat odour by host‐seeking insects. Biological Reviews, 92, 1241–1249. 10.1111/brv.12281 27145528

[ece34534-bib-0055] Wickham, H. (2016). ggplot2: Elegant graphics for data analysis. New York, NY: Springer.

[ece34534-bib-0056] Wiklund, C. (1975). The evolutionary relationship between adult oviposition preferences and larval host plant range in *Papilio machaon* L. Oecologia, 18, 185–197. 10.1007/bf00345421 28308676

[ece34534-bib-0057] Wise, M. J. , Yi, C. G. , & Abrahamson, W. G. (2009). Associational resistance, gall‐fly preferences, and a stem dimorphism in *Solidago altissima* . Acta Oecologica, 35, 471–476. 10.1016/j.actao.2008.12.005

[ece34534-bib-0058] Yang, C. H. , Belawat, P. , Hafen, E. , Jan, L. Y. , & Jan, Y. N. (2008). *Drosophila* egg‐laying site selection as a system to study simple decision‐making processes. Science, 319, 1679–1683. 10.1126/science.1151842 18356529 PMC2581776

[ece34534-bib-0059] Zhang, Y. V. , Aikin, T. J. , Li, Z. , & Montell, C. (2016). The basis of food texture sensation in *Drosophila* . Neuron, 91, 863–877. 10.1016/j.neuron.2016.07.013 27478019 PMC4990472

