# Supplementary material for: Sensory deficiencies affect resource selection and associational effects at two spatial scales
Source: Ecol Evol. 2018 Oct 3;8(21):10569–77. doi: 10.1002/ece3.4534 (PMC6238129; doi:10.1002/ece3.4534)
Supplement: Supplementary file 1 [file ECE3-8-10569-s001.pdf]

Sensory deficiencies affect resource selection and associational effects at two spatial scales

Supplementary Information

Author affiliation:

Thomas A. Verschut* (http://orcid.org/0000-0003-0130-6485)

Department of Ecology, Environment and Plant Sciences, Stockholm University, 106 91 Stockholm, Sweden. Email: thomas.verschut@su.se

***Corresponding author**

Brian D. Inouye (http://orcid.org/0000-0003-3994-2460)

Department of Biological Science, Florida State University, Tallahassee, Florida 32306, United States of America. Email: bdinouye@bio.fsu.edu

Peter A. Hambäck (http://orcid.org/0000-0001-6362-6199)

Department of Ecology, Environment and Plant Sciences, Stockholm University, 106 91 Stockholm, Sweden. Email: peter.hamback@su.se

Table S1. Summary of likelihood ratio test (χ^2) for the model testing the differences in the relative oviposition patterns among the wild type (*w^{1118}*), olfactory deficient (*$Orco^2$*) and gustatory deficient (*$Poxn^{\Delta M22\text{-}B5}$*) *Drosophila melanogaster* strains. *df* gives the between-groups degrees of freedom followed by the within-groups degrees of freedom (i.e. residuals).

**Model**	**Deviance**	**Factors**	**χ^2**	***df***	***P***
w^{1118} x *Orco2* x *$Poxn^{\Delta M22\text{-}B5}$*	10623.7	Resource Frequency	23.13	1, 2854	**<0.001**
		Resource distribution	0.08	1, 2854	0.779
		Resource type	655.34	1, 2854	**<0.001**
		Strain	19.18	2, 2854	**<0.001**
		Resource Frequency *x* Resource distribution	0.07	1, 2854	0.797
		Resource Frequency *x* Resource type	115.24	1, 2854	**<0.001**
		Resource Frequency *x* Strain	16.57	2, 2854	**<0.001**
		Resource distribution *x* Resource type	0.43	1, 2854	0.512
		Resource distribution *x* Strain	0.74	2, 2854	0.689
		Resource type *x* Strain	630.10	2, 2854	**<0.001**
		Resource Frequency *x* Resource type *x* Strain	14.35	2, 2854	**<0.001**
		Resource distribution *x* Resource type *x* Strain	1.09	2, 2854	0.581
		Resource Frequency *x* Resource distribution *x* Resource type *x* Strain	14.05	5, 2854	**0.015**

Table S2. Summary of likelihood ratio test (χ^2) for the relative oviposition patterns of the wild type (*w^{1118}*), olfactory deficient (*$Orco^2$*) and gustatory deficient (*$Poxn^{\Delta M22\text{-}B5}$*) *Drosophila melanogaster* strains separately. *df* gives the between-groups degrees of freedom followed by the within-groups degrees of freedom (i.e. residuals).

**Type**	**Deviance**	**Factors**	χ^2	***df***	***P***
Wild type	3420.0	Resource Frequency	42.93	1, 950	<**0.001**
(*w^{1118}*)		Resource distribution	0.26	1, 950	0.609
		Resource type	650.71	1, 950	<**0.001**
		Resource Frequency *x* Resource distribution	1.06	1, 950	0.302
		Resource distribution *x* Resource type	0.01	1, 950	0.945
		Resource Frequency *x* Resource type	64.12	1, 950	<**0.001**
		Resource Frequency *x* Resource distribution *x* Resource type	3.47	1, 950	0.063
Olfactory deficient (*$Orco^2$*)	3355.5	Resource Frequency	22.18	1, 950	<**0.001**
		Resource distribution	0.01	1, 950	0.971
		Resource type	621.09	1, 950	<**0.001**
		Resource Frequency *x* Resource distribution	0.29	1, 950	0.591
		Resource distribution *x* Resource type	0.84	1, 950	0.359
		Resource Frequency *x* Resource type	44.17	1, 950	<**0.001**
		Resource Frequency *x* Resource distribution *x* Resource type	7.64	1, 950	**0.005**
Gustatory deficient (*$Poxn^{\Delta M22\text{-}B5}$*)	3837.1	Resource Frequency	7.90	1, 950	**0.005**
		Resource distribution	1.01	1, 950	0.316
		Resource type	8.56	1, 950	**0.003**
		Resource Frequency *x* Resource distribution	0.01	1, 950	0.921
		Resource distribution *x* Resource type	0.08	1, 950	0.769
		Resource Frequency *x* Resource type	20.71	1, 950	<**0.001**
		Resource Frequency *x* Resource distribution *x* Resource type	0.98	1, 950	0.322

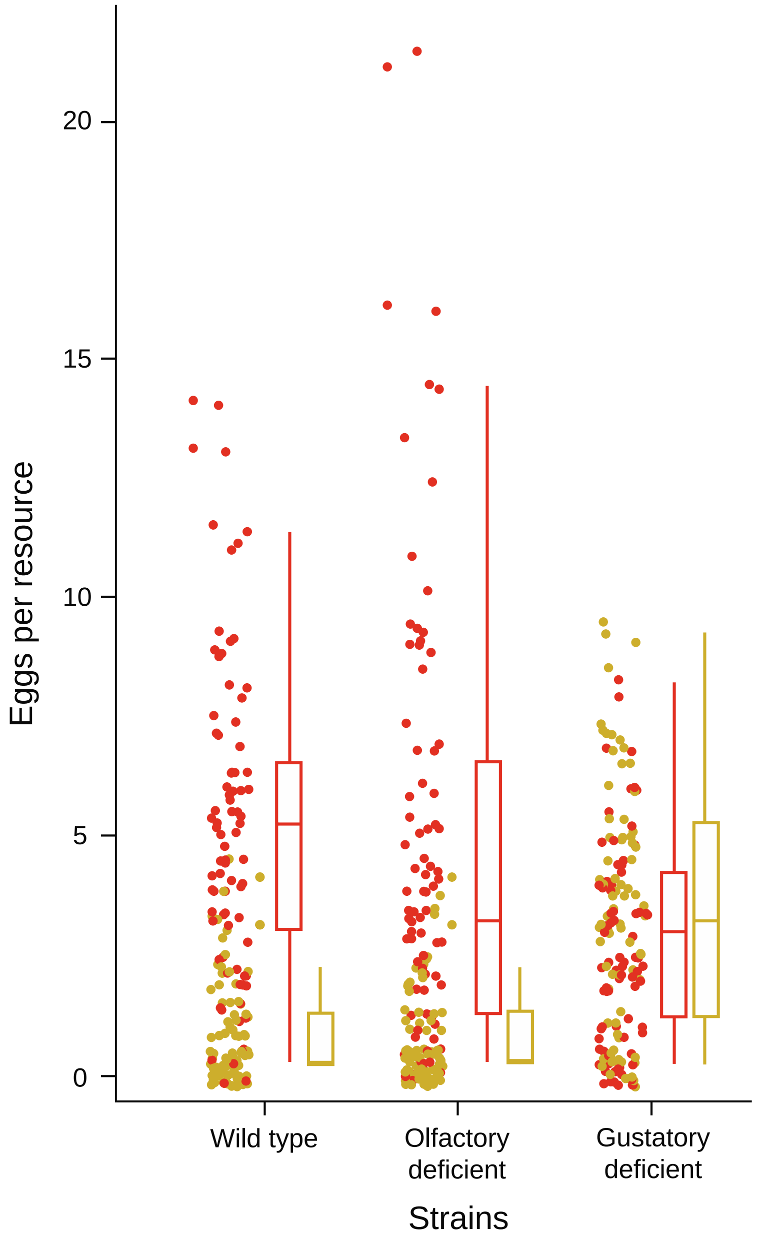

Fig. S1. The oviposition behavior of the wild type (w^{1118}), olfactory deficient (*Orco*^{2}), and gustatory deficient (*Poxn*$^{\Delta M22\text{-}B5}$) *Drosophila melanogaster* strains in the additional treatment used in the estimation of the resource selection coefficients. This treatment had an overall 4:4 ratio, but the oviposition substrates were arranged such that there were 3:1 and 1:3 ratios of each resource type within each patch. Each point in the graph represents the number of eggs laid by an individual female either on an apple (red) or banana (yellow) oviposition substrate. The points are jittered horizontally and the boxplots next to the data point show the spread of the data for apple (red) or banana (yellow).
